# Protest of doctors: a basic human right or an ethical dilemma

**DOI:** 10.1186/1472-6939-15-24

**Published:** 2014-03-10

**Authors:** Imran Naeem Abbasi

**Affiliations:** 1 Research fellow, Department of Community Health Sciences, Aga Khan University, Stadium Road, P.O. Box-3500, Karachi, Pakistan

**Keywords:** Ethical dilemma, Doctors’ strikes, Pakistan, Service structure, Lack of security, Low pay, Socialized medicine, Utilitarian theory, Brain drain

## Abstract

**Background:**

Peaceful protests and strikes are a basic human right as stated in the United Nations’ universal declaration on human rights. But for doctors, their proximity to life and death and the social contract between a doctor and a patient are stated as the reasons why doctors are valued more than the ordinary beings. In Pakistan, strikes by doctors were carried out to protest against lack of service structure, security and low pay. This paper discusses the moral and ethical concerns pertaining to the strikes by medical doctors in the context of Pakistan. The author has carefully tried to balance the discussion about moral repercussions of strikes on patients versus the circumstances of doctors working in public sector hospitals of a developing country that may lead to strikes.

**Discussion:**

Doctors are envisaged as highly respectable due to their direct link with human lives. Under Hippocrates oath, care of the patient is a contractual obligation for the doctors and is superior to all other responsibilities. From utilitarian perspective, doctors’ strikes are justifiable only if there is evidence of long term benefits to the doctors, patients and an improvement in service delivery. Despite that, it is hard to justify such benefits against the risks to the patients. Harms that may incur to the patients include: prolongation of sufferings, irreversible damage to health, delay in treatment, death, loss of work and waste of financial resources.

In a system of socialized medicine, government owing to greater control over resources and important managerial decisions should assume greater responsibility and do justice to all stakeholders including doctors as well as patients. If a doctor is underpaid, has limited options for career growth and is forced to work excessively, then not only quality of medical care and ability to act in the best interests of patients is adversely affected, it may also lead to brain drain.

**Summary:**

There is no single best answer against or in favor of doctors’ industrial action. The author calls for the debate and discussion to revitalize the understanding of the ethical predicaments of doctors’ strikes with patient care as the priority.

## Background

Around the world, strikes and protests are held to condemn violation of basic human rights, to strongly put forward one’s point of view to the authorities or for fulfillment of the specific needs. According to the United Nations’ universal declaration on human rights articles 19 and 20, right to freedom of expression and peaceful assembly is a basic human right [1] and the governments are responsible for ensuring that everyone can exercise it without fear of intimidation or violence [2].

But for doctors, right to strike and protest is viewed from different perspective known as the social contract. A doctor enters the social contract with his patient after swearing the Hippocrates oath. According to this oath, he/she swears to act in the highest interest of his patient and keep the health and life of his patient a priority above everything [3]. Due to their proximity to life and death situations, strikes by doctors are perceived as an ethical misconduct by the society. Young doctors in Pakistan have been carrying out such strikes against low wages, lack of security and service structure [4-6]. The purpose of the article is to discuss and debate the role of socialized medicine and utilitarian view, the ethical principle that is followed to judge the strike by doctors. The article describes the circumstances and sequence of events that led to the strike. Ethical predicaments arising as a result of the strike, the role of all stakeholders including government and doctors in strike and the impact of strikes on patients are discussed in detail. The author argues that if a doctor is underpaid, has no options of career growth and is forced to work excessively, then not only do the quality of medical care and the ability to act in the best interests of patients get adversely affected, it can also lead to brain drain. In the end, the author has raised some key questions for open debate with the intent of developing a common understanding about the ethical aspects of doctors’ strike and to minimize the loss to patients.

### Protest of doctors in Pakistan

Interns and postgraduate doctors (PGs) in the public health sector of Pakistan have continued to work under adverse conditions in the form of low pay, long working hours and unsuitable work environment. Due to lack of planning and poor governance, there were not enough vacancies to accommodate all the postgraduate trainees on payroll in public sector hospitals. As a result, they had to work without a pay for the sake of completion of the postgraduate training. Pakistan is among the countries where physician density per 1000 population is less than 1 [7]. Therefore while training completion is a necessity, given the critical workforce deficiency trainees have served as an important human resource for the health care system. Later, College of physicians and surgeons Pakistan (CPSP) [8], a regulatory body for postgraduate accreditation and training imposed a ban on unpaid training. Though the ban prevented such misuse of post-graduate trainees to some extent, but unpaid training still continues and problems of low pay for young doctors like interns and PGs have remained unresolved. Given these circumstances, an informal body namely Young Doctors’ Association (YDA) stepped forward for negotiations with the government for resolution of the problems of young doctors. Some of the key points put forward by YDA are as following [9,10]:

• Special salary package for the doctors at all levels from interns (house officers) to postgraduate trainees

• Provision of security in all emergency departments and indoor hospital units

• Introduction of a service structure for doctors working in the public health sector

Among these, greater emphasis was put on introduction of service structure. Service structure exists in various other professions enabling individuals to have ample opportunities for career growth and pay raise. On the contrary, this remains an ignored aspect in the health policy of Pakistan [11]. After prolonged negotiations, government agreed to give some raise in the salaries of young doctors [12] but issues of provision of security and service structure remained unaddressed despite several rounds of negotiations. Finally the continued ignorance led to token strikes by doctors in order to more strongly convey their message to the authorities. But apathy of the authorities acted as fuel on fire with escalation of the issue in the form of suspension of out-patient services [4,5]. In-patient services for admitted patients and emergency services were kept operational to prevent damage to health and to avoid loss of lives. Government rather acted in an unexpected manner. Law enforcement agencies were called in order to end the protests. Camps where peaceful protests were being carried out were uprooted and several peacefully protesting doctors were physically tortured and arrested. Many of the doctors, who were part of the protests, were suspended from their duties.

The issue of doctors’ protests was highlighted in print and electronic media with more focus on patients’ sufferings [6].

## Discussion

### Utilitarian view

Doctors are envisaged as highly respectable in every society because of their direct link with human lives. Due to their proximity to life and death, society judges them by standards higher than those of ordinary humans. Their role in alleviating the pain and misery of the people entitles them as ‘the Massiahas’ (the saviors) of mankind by the society. Under Hippocrates oath, care of the patient becomes a contractual obligation for the doctors and is superior to all other responsibilities [3]. Utilitarian perspective views doctors’ strike justifiable only under the circumstances where there is evidence of long term benefits to doctors, improvement in service delivery and when those in need of health care will seek the greatest benefit out of the strike [3,13]. But the formula becomes increasingly complex when the benefits of the strike are to be weighed against the risks to the patients. Due to the nature of the damage that might be inflicted to the patients following the strike, measuring benefits of strike becomes rather tricky.

The ethical obligation of a person to join the profession of medicine and become a doctor can be debated. That is to say, what influences an individual to become a doctor, whether these are societal needs where there is a dearth of skilled personnel or if it’s an open choice without any moral obligations? The utilitarian theory, however debates that when a person finally decides to carry out a particular job, then he/she must obey its ethical obligations [3]. That is, when a person decides to become a doctor, then he/she implicitly as well explicitly enters the social contract with his/her patients.

### Socialized medicine

Socialized medicine is aimed at providing care at minimal cost to the majority of the population. In socialized medicine, responsibility of patient care is shared by the doctor, the health care institution and the government. The government provides funding, physical infrastructure and manpower to the institutions, hospitals in this case, that are responsible for ensuring the continuity of care and operational efficiency. In this kind of set up, if any one of the entities fails in fulfilling its commitment, the consequences are borne by the remaining entities. For example if the government fails in maintaining the job satisfaction of its employees or fulfilling its commitments as happened in the case of doctors’ protest in Pakistan, then the sanctity of the other two entities may also be adversely affected. But, in case of government’s failure, doctors’ decision to strike could still be regarded as an individual choice i.e. whether to go on strike or to obey the social contract. The questions that arise about the morality of the strike are: despite the government’s failure, is going on strike a collective responsibility of all three entities? Should doctors continue to be obliged by their social contract and as a result suffer themselves? Who should be held accountable for the patients’ sufferings?

### Case of doctors’ sufferings

A doctor’s career path is a hard choice in Pakistan. Only after spending a minimum of 10 years of education and training, a doctor earns a reputable postgraduate degree which is a considerably long duration. Working conditions of doctors particularly in public sector hospitals are a pity. Low pay, extended working hours and lack of safety and security are some of the common issues that doctors face in every public sector hospital. Unfortunately, lack of good governance is a hindrance; due to which health is not a priority area for policy makers and planners. Because of the same reason, Pakistan merely spends 2.5% of it's gross domestic product (GDP) on health, a percentage lower than any neighboring developing country including India and Bangladesh [14]. Should health be a priority subject in the agenda, prioritizing health issues including provision of better working conditions and service structure should not prove a hard bone to swallow.

Yet, the question that whether such adverse working conditions of doctors justify their strike action has no straight forward yes or no answer. Suspension of care provision can be an effective way of pressurizing the government to realize the consequences of a strike on the overall health care system. But whether it is the government’s fault in creating such conditions, or a way of doctors to embarrass the government, both circumstances lead to breach of the moral contract between doctors and patients. The ultimate result of these situations is health care denied to the patients. Viewing doctors’ protest from the perspective of socialized medicine might be perceived as a consequence of government’s negligence, but according to utilitarian theory and the Hippocrates oath it does not void the moral duty of doctors to prioritize patients’ health.

### Patients’ sufferings

Historically, strikes by doctors were kept limited to non-emergency cases while keeping the emergency services functional. Such types of strikes were carried out in Israel, Australia, Tanzania and more recently in the UK. In Israel, an alternate system called fee for service was established to deal with outpatients to ensure continuity of care during strike [3,12,15,16]. In the UK, it was called as ‘urgent and emergency care model’ where all non-emergency surgeries, investigations, out-patient consultations and routine procedures were kept suspended [17]. Park et al. (2013) argues that doctors can protest given that any emergency care required is urgently provided. However, the definition of emergency as defined by doctors may differ from the general perception. Delaying care to non-emergency cases may turn their condition into preventable emergency cases. Therefore, this mode of the strike also bears moral repercussions for doctors. Strikes may impact patients in the form of an increase in severity of the medical condition, prolongation of sufferings, irreversible damage to health or loss of life, delay in treatment or unwanted drug interruptions, loss of work and waste of money on transportation [18].

Suspension of public sector health care services turns the flow of the patients to the private sector. Public health services are relatively cheap compared to private sector and therefore people belonging to low socioeconomic status may not be able to receive health care due to issue of cost. Moreover, private health sector’s capacity may not be adequate to meet the requirements of the population in times of strike adding to the misery of those in most need of health care.

#### *Reflections*

The issue seems to have no straight right or wrong direction. Yet it invites debate over the key points: i) responsible role of government in preventing strikes and the consequent system failure ii) role of doctors in ensuring continuity of care during strikes and minimizing its impact on those in most need of health care.

First, since the social contract between doctor and patient is not considered void under any circumstances, strikes by doctors seem to raise ethical concerns about their professional conduct. Here it is vital to consider that doctors in their entirety are human beings having similar emotions, feelings and more importantly the needs as those of a common man. Bounding them with social contract does not eliminate these basic human characteristics. Rather it puts an added burden in the form of social responsibility tied to their profession which is the case of doctors’ strikes in Pakistan. Their working conditions, low pay and lack of opportunities for career growth continued to fuel the frustration. Therefore, while it is expected from doctors to obey the social contract, consistently ignoring their basic needs may lead to circumstances that then manifest in the form of so called undesired outcomes. Forcing doctors to work under adverse working condition can lead to demotivation and demoralization that may compromise the quality of care provided to the patients.

Second, enjoying an optimum level of health is the basic right of every individual and is a constitutional responsibility of the state. The state is responsible for ensuring the adequacy of resources both financial and human, and proper resource allocation. Lack of seriousness and timely interventions into the issues such as strikes ultimately ends in patients’ sufferings. Moreover, in the system of socialized medicine government is the supreme authority since it has the control of resources and important management decisions related to health (care) system. Therefore, government has the responsibility to do the justice to the system and to all of its stakeholders. Government also needs to adopt a responsible role regarding how to tackle these strikes by doctors. Use of physical violence and force to end the strikes can not only result in the escalation of the issue but it shows lack of interest on the part of government in addressing the various problems of the doctors including job satisfaction and career growth. This ultimately has led to a situation of brain drain where doctors are fleeing abroad for better future option. This is a cause of great concern in a country already suffering from a serious shortage of health workforce [6]. Considering these circumstances, is there a need to review our understanding of the ethical guidelines regarding who needs to take the responsibility for the patients’ sufferings? Or should one continue to pursue the path of Hippocratic Oath irrespective of the root causes of the strike? Author calls for the debate and discussion over the questions raised, with an intent to come to a greater understanding of the ethical issues of doctors’ strike. The ultimate goal of such a debate is to strive for an effective health system with a mutual respect for its stakeholders (government, doctors and patients).

Finally, the impartial role of print and electronic media is important while reporting any issue for the insight of public. People should have access to information and view point of all stakeholders which will help them in deciding the legitimacy of the situation. Media can act as an important tool in developing an understanding among all the stakeholders during strike by verbalizing each stakeholder’s point of view, should it wish to do so.

## Summary

Doctors’ strikes present an ethical dilemma because of the pivotal role of doctors in preventing disease and injury to the human body. Doctors are viewed and judged by higher standards than the ordinary people. A doctor is under social contract and moral obligation to keep patient’s health and life as a priority. Going on strike constitutes a breach of such contract. Utilitarian view pronounces strike as ethical only under the condition when such action carries greater good for the doctors and to society than the loss occurring as a result of denial of health services to the patients during the strike. Despite the fact that socialized medicine being practiced in several developing countries in which health care is the joint responsibility of the state, the hospital and the doctor, the doctors’ action of going on strike carries greater ethical predicaments for themselves. There is no single best answer against or in favor of doctors’ industrial action. However, in a system where socialized system of medicine is widely practiced, government being in charge of resources and management decisions should assume greater responsibility in faith of the greater good of all stakeholders including doctors and patients. Working conditions of doctors in developing countries in particular demand a contextual analysis of the situation.

Though, keeping the emergency services functional while on strike is a responsible act in favor of those severely sick, denials of non-urgent care can turn such cases into preventable emergency conditions. Either way, the ultimate bearers of the consequences of the strike are those in most need of health care.

## Abbreviations

GDP: Gross domestic product; CPSP: College of physicians and surgeons Pakistan.

## Competing interests

The author declares that he has no competing interests.

## Author’s information

Imran Naeem Abbasi has completed his residency in community medicine and is currently working as research fellow in the department of Community Health Sciences, the Aga Khan University Karachi. His area of interest is health systems with special focus on social determinants of health and policy issues.

## Pre-publication history

The pre-publication history for this paper can be accessed here:

http://www.biomedcentral.com/1472-6939/15/24/prepub
